# Hepatitis A virus exhibits a structure unique among picornaviruses

**DOI:** 10.1007/s13238-014-0103-7

**Published:** 2014-10-31

**Authors:** Ling Zhu, Xiaoxue Zhang

**Affiliations:** 1 National Laboratory of Macromolecules, Institute of Biophysics, Chinese Academy of Science, Beijing, 100101 China; 2 Division of Structural Biology, University of Oxford, The Henry Wellcome Building for Genomic Medicine, Headington, Oxford, UK; 3 Higher Education Press, Beijing, 100029 China

Hepatitis A, which is a major global health problem (Nainan et al., 2006), is caused by hepatitis A virus (HAV). Approximately 1.4 cases of hepatitis A have been reported annually. HAV belongs to the family of *Picornaviridae* and genus *Hepatovirus*, which is a sole member of this family. HAV has radically different properties from those of other picornaviruses. First, HAV is extremely resistant to degradation caused by environmental conditions, which include thermal denaturation (i.e., HAV survives at 70°C for up to 10 min), acid treatment (i.e., pH 1 for 2 h at room temperature), 20% ether and chloroform, and detergent inactivation (i.e., HAV survives at 37°C for 30 min in 1% SDS). Second, the HAV has a highly de-optimized codon usage and grows slowly in a tissue culture. Third, VP1-2A or PX, which is a 67-residue C-terminal extension of VP1, has been implicated in the particle assembly (Graff et al., 1999) of HAV; further, particles contain an extension shroud in a host membrane to produce enveloped viruses (Feng et al., 2013). Fourth, the residues of putative VP4 in HAV is extremely small (~23 residues), and the presence of this protein in particles remains unclear (Martin and Lemon, 2006). Finally, although TIM-1 has been identified as a cellular receptor of HAV years ago (Kaplan et al., 1996), TIM-1 has been recently considered a common enhanced receptor for some enveloped viruses (Moller-Tank et al., 2013). Meanwhile, a mechanism explaining the movement of virus to the liver, which is its principal site of replication, is still uncertain.

Recently, structures of a mature virion and an empty capsid assembly intermediate of HAV were determined by X-ray crystallography (Wang et al., 2014). Determination of these structures represents a significant achievement because several researchers have attempted to study this virus without success. Moreover, determination of the structures significantly extended our understanding of structure variability within the *Picornavirus* family. VP4, which is a small viral protein, was first detected in HAV mature virions. The external surface of HAV exhibits fewer features than those observed in enteroviruses and cardioviruses (Wang et al., 2012; Ren et al., 2013; Luo et al., 1987); this surface appears as a facetted triakis icosahedron. The smooth particle surface is devoid of depressions because of relatively short loops that correspond to BC, GH loop of VP1, and EF loops of VP2; and this depression might correspond to receptor binding sites in enteroviruses (Dang et al., 2014). The HAV does not contain a continuous hydrophobic pocket in VP1 and has no pocket factor, however, can withstand a remarkably high temperature and low pH. These structural features indicate that the HAV probably uncoats via a novel mechanism, which is different from those utilized by other picornaviruses (De Colibus et al., 2014). The most striking structural characteristic is its VP2 “domain swap” that creates a link between neighboring pentamers. This characteristic is not exhibited by other known picornavirus structures; however, a distantly related insect virus structure (cricket paralysis virus) has exhibited this feature (Tate et al., 1999). The re-writing of VP2 also may reflect a fundamental difference in the mechanism of HAV uncoating. An enterovirus utilizes an umbilicus composed of an amphipathic N-terminal helix of VP1 and VP4 to transfer a viral RNA genome (Panjwani et al., 2014); these procedures are triggered by the functional receptors (or low pH) that mediate expulsion of a viral pocket factor (Tuthill et al., 2010), convert a mature virion into its “expanded form” (Ren et al., 2013; Butan et al., 2014). The HAV is expected to adopt a novel uncoating mechanism because it does not contain a canyon or a pocket factor and proves to be robust at very low pH and remarkably high temperature. A structure-based phylogeny analysis places HAV between typical picornaviruses and insect viruses. The inexplicable properties of HAV that moves cell-to-cell by transcytosis and potentially distinctive uncoating mechanism reflect its position as a link between “modern” picornaviruses and the more “primitive” precursor insect viruses.

Although the structure of HAV presents significantly different properties compared with other previously characterized viruses, reveals the phylogenic relationship between typical picornaviruses and insect viruses and also indicates a novel uncoating mechanism, several important biological parameters, such as the entry of HAV and release of an RNA genome, still need to be clarified.
